# Exposure to Novel Parainfluenza Virus and Clinical Relevance in 2 Bottlenose Dolphin (*Tursiops truncatus*) Populations

**DOI:** 10.3201/eid1403.071250

**Published:** 2008-03

**Authors:** Stephanie Venn-Watson, Rebecca Rivera, Cynthia R. Smith, Jeremiah T. Saliki, Shannon Caseltine, Judy St. Leger, Pam Yochem, Randall S. Wells, Hendrik Nollens

**Affiliations:** * US Navy Marine Mammal Program, San Diego, California, USA; ‡ G2 Software Systems, Inc., San Diego, California, USA; ‡ Hubbs-SeaWorld Research Institute, San Diego, California, USA; § Oklahoma State University, Stillwater, Oklahoma, USA; ¶ SeaWorld, San Diego, California, USA; # Chicago Zoological Society c/o Mote Marine Laboratory, Sarasota, Florida, USA; ** University of Florida, Gainesville, Florida, USA; 1 Current affiliation: University of Georgia, Athens, Georgia, USA.

**Keywords:** dolphin, marine mammal, parainfluenza virus, seroepidemiologic studies, *Tursiops truncatus*, research

## Abstract

Parainfluenza virus (PIV) is a leading cause of respiratory infections in humans. A novel virus closely related to human and bovine parainfluenza viruses types 3 (HPIV-3 and BPIV-3), named *Tursiops truncatus* parainfluenza virus type 1 (TtPIV-1), was isolated from a dolphin with respiratory disease. We developed a dolphin-specific ELISA to measure acute- and convalescent-phase PIV antibodies in dolphins during 1999–2006 with hemograms similar to that of the positive control. PIV seroconversion occurred concurrently with an abnormal hemogram in 22 animals, of which 7 (31.8%) had respiratory signs. Seroprevalence surveys were conducted on 114 healthy bottlenose dolphins in Florida and California. When the most conservative interpretation of positive was used, 11.4% of healthy dolphins were antibody positive, 29.8% were negative, and 58.8% were inconclusive. PIV appears to be a common marine mammal virus that may be of human health interest because of the similarity of TtPIV-1 to BPIV-3 and HPIV-3.

## Abstract 2

Evidence of PIV exposure was detected in free-ranging and managed dolphin populations living along 2 US coastlines.

Parainfluenza viruses (PIVs) are often associated with respiratory illness in terrestrial mammals, including croup in humans (*1*), kennel cough in dogs (*2*), and bovine respiratory disease in cattle (*3*). A novel PIV tentatively named *Tursiops truncatus* parainfluenza virus type 1 (TtPIV-1) was cultured from lung tissue in an Atlantic bottlenose dolphin (*T. truncatus*) (*4*). This animal had respiratory disease including laryngitis, tracheitis, and bronchointerstitial pneumonia with mild to moderate growth of *Candida glabrata.*

Phylogenetic analyses of 2 genomic fragments of TtPIV-1 showed that the virus strain was monophyletic with, but genetically distinct from, bovine parainfluenza virus 3 (BPIV-3) strains and human parainfluenza type 3 (HPIV-3) (*4*). BPIV-3 is an effective antigenic stimulator in humans and is used in human vaccines that protect against HPIV-3 (*5*–*7*). TtPIV-1 may provide similar protection in humans. Dolphins have been recognized as useful marine ecosystem sentinels (*8*), and changes in marine PIV may reflect changes in terrestrial PIV.

ELISAs have been characterized as the most sensitive diagnostic tool to identify rising titers due to PIV-associated respiratory illness in humans (*9*). Although ELISA is the ideal tool for identifying infections caused by PIV, high levels of antigenic cross-reactivity among various PIV subfamilies and other closely related viruses hinder the ability for ELISA to determine which type of PIV has infected an animal (*10*,*11*). In wild marine mammal populations, ELISA-based serosurveys for suspected viral or bacterial pathogens are common (*12*–*14*). Limitations of these studies include unknown health status of animals or lack of paired samples that can differentiate exposures from active infections.

The US Navy Marine Mammal Program (MMP) manages a population of bottlenose dolphins that live in San Diego Bay, California. These animals are provided high-quality medical and preventive care throughout their lifetime. Standardized health data and voluntary blood samples are collected routinely, uniquely enabling MMP to amass routine physiologic information on dolphins living in a marine environment at all age stages. Since 1988, health assessments have been conducted by the Chicago Zoological Society and collaborators on a free-ranging, resident coastal population of bottlenose dolphins in Sarasota Bay, Florida, 2,500 miles away, as part of the world’s longest running study of wild dolphins. Serum samples from this presumably healthy population are archived for use in retrospective health assessments.

An indirect, dolphin-specific PIV-antibody ELISA was developed and applied to archived serum samples collected from MMP dolphins in San Diego (1999–2006) and healthy, free-ranging dolphins living near Sarasota (2004–2005). We used this ELISA to assess the clinical relevance of PIV exposure and seroconversion in bottlenose dolphins living along US coasts.

## Methods

The MMP is routinely reviewed by an Institutional Animal Care and Use Committee (IACUC) and Navy Bureau of Medicine; the MMP is accredited by the Assessment and Accreditation of Laboratory Care International. All sample collection protocols for the Sarasota wild dolphin population were approved by the University of Florida IACUC (IACUC no. C233).

### San Diego, California

Blood samples from MMP dolphins were initially collected by venipuncture from animals trained either to present their tail for sampling in the water or to rest on a foam mat during a routine physical examination out of the water. Samples were collected from the caudal peduncle vein by using a 20- or 21-gauge, 1.5-inch Vacutainer needle (Becton Dickinson Vacutainer Systems, Rutherford, NJ, USA) or from a fluke vein by using a 21-gauge, 1-inch butterfly needle. Blood was collected into a Vacutainer serum separator tube or a Vacutainer EDTA (K_3_) tube for serum chemistries and complete blood counts, respectively.

Samples for chemistry analysis were centrifuged within 2 h of collection. Centrifugation was performed at 3,000 rpm at 21°C for 10 min. Fibrin clots were removed, and serum was transferred to a 5-mL plastic submission tube. Whole blood was collected in EDTA Vacutainer tubes. All samples were sent on wet ice by courier to Quest Diagnostic Laboratories in San Diego.

Automated hematologic analyses were conducted by Quest Diagnostic Laboratories with the Coulter LH 1500 Series (Beckman Coulter, Inc., Fullerton, CA, USA). The Fisherbrand Dispette 2 (Fisher Scientific, Pittsburgh, PA, USA), correlating with the Westergren method, was used in house to determine 60-min erythrocyte sedimentation rates (ESRs) from 1 mL EDTA whole blood. Remaining serum from these samples was archived at –80°C at the MMP facility at the time of initial blood collection.

Upon completed development of the PIV-antibody ELISA, the archived, frozen serum samples were shipped frozen overnight to the laboratory for PIV-antibody analysis. Total leukocyte count, absolute neutrophils, absolute lymphocytes, absolute monocytes, absolute eosinophils, and ESR results were incorporated into the retrospective PIV seroprevalence study and linked to animal age, sex, clinical signs, and PIV-antibody ELISA results.

### Sarasota Bay, Florida

Blood samples from free-ranging dolphins were obtained as part of a long-term health assessment conducted near Sarasota, involving a multigenerational resident population of ≈150 dolphins (*8*). Most of these dolphins are recognizable; are of known age, gender, and maternal lineage; and medical histories have been recorded for many years. Small groups of dolphins were encircled with a 5,000-m long × 5-m deep seine net in shallow water (<1.8 m). Each dolphin was shaded, kept cool and wet, and carefully monitored for signs of discomfort by 1 to 3 veterinarians experienced with cetaceans. The veterinary staff monitored the dolphin’s respiration rate and quality, responsiveness to external stimuli, mental alertness, skin temperature, and heart rate to evaluate the animal’s comfort level and immediate health status. Blood samples were drawn from the ventral fluke vasculature by a 19-gauge × ¾-inch butterfly catheter with a multisample adaptor (Becton Dickinson), which allowed the blood collection tubes to be filled directly from the venipuncture set.

### ELISA Development

#### Antigen Production

Archived TtPIV-1 was propagated in BSC40 cells as previously described (*15*). Uninfected BSC40 cells were cultured as negative controls. When >90% of the infected monolayers showed cytopathic effect (CPE), all infected and uninfected cultures were harvested and centrifuged for 15 min at 1,500 rpm, and the supernatant media was removed. The remaining cell pellets were freeze-thawed 3× and centrifuged for 15 min at 3,000 rpm. The cell pellet was discarded, and the supernatant cell lysates were pooled for use as antigen in the ELISA. The protein concentration of the infected and uninfected cell lysates was determined by using a modified Bradford assay for ELISA, after which the cell lysates were diluted in phosphate-buffered saline (PBS) to the desired coating concentration.

#### Optimization of ELISA Parameters

A serum sample collected postmortem from the case dolphin was used as the positive reference serum. A negative reference serum sample was collected from an immunologically naive neonate bottlenose dolphin that had not yet nursed. The positive and negative reference serum samples were used to optimize the ELISA conditions. All assay parameters were varied (working volume 50–100 μL; coating concentration 1–20 μg/mL; serum dilution 1:50–1:400; developing time 15–60 min), and the assay conditions with the highest ratio of the optical density at 405 nm (OD_405_) of positive reference serum sample to the OD_405_ of negative reference serum sample were selected. The conditions of the optimized ELISA protocol were as follows. Wells of a high protein-binding microplate (Nunc Maxisorp, Fisher Scientific) were coated with 50 μL of infected or uninfected cell lysates at 5 μg/mL in PBS and were left to adsorb overnight at 4°C. After this and each subsequent step, all wells were washed 3× with PBS with 0.05% Tween by using an automated EL 404 microplate washer (Biotek Instruments, Winooski, VT, USA). After washing, all wells were blocked with 300 μL of Superblock blocking buffer (Pierce, Rockford, IL, USA) in PBS with sodium azide (PBS/Az), after which the dolphin serum samples were applied (1:500 in 1% bovine serum albumin [BSA] in PBS/Az). All sera were applied in triplicate to wells that were coated with either infected or uninfected cell lysate antigen. The positive and negative control sera were included on each plate. A biotinylated monoclonal antibody specific for bottlenose dolphin IgG (*15*) was used at a concentration of 5 µg/mL (in 1% BSA in PBS/Az) as the secondary reagent for the detection of bound antibodies. Each step of the ELISA was left to incubate with gentle agitation (Nutator; Adams, Fisher Scientific) for 1 h at ≈22°C. Finally, 1.0 mg/mL p-nitrophenyl phosphate (Sigma, St. Louis, MO, USA) substrate was added. The OD_405_ was recorded 60 min after addition of the substrate by using a Spectramax 250 microplate reader (Molecular Devices, Sunnyvale, CA, USA). For analysis, the mean OD_405_ of the triplicate readings on the uninfected antigen was subtracted from the mean OD_405_ of the triplicate readings by using the infected antigen. All results were presented as OD_405_ ratios, defined as the OD_405_ reading of the unknown samples divided by the OD_405_ reading of the positive control sample for that plate.

### Cut-off Values, Definitions, and Data Analysis

All data were analyzed with SAS software (Release 8e; SAS Institute, Inc., Cary, NC, USA). p values <0.01 were defined as significant.

#### PIV Seroconversion

Archived hematologic and clinical observation data (1999–2006) were mined to identify dolphins with hemograms similar to that of the positive control animal, including a neutrophilic, monocytic leukocytosis or a high ESR, as defined by MMP reference ranges (*16*). Archived serum samples collected within 60 days before, during, and <90 days after an inflammatory hemogram or clinical illness from suspected PIV cases were subsequently analyzed for PIV antibodies with the dolphin-specific ELISA. PIV seroconversion was defined as a >4-fold increase in PIV antibody OD_405_ level within a 3-month period.

Analyses were conducted to describe frequencies of abnormal clinicopathologic values and clinical signs among PIV seroconversion cases from 60 days before the highest OD_405_ ratio to 30 days after the highest OD_405_ ratio. Median clinicopathologic blood values were calculated among PIV seroconversion cases by using the serum sample with the highest leukocyte count from each identified animal (e.g., the blood sample most likely representing the most severe phase of disease).

#### Seroprevalence among Healthy Populations

Serum samples collected during July to December 2006 from 58 MMP bottlenose dolphins in San Diego and samples collected during 2003–2005 from 56 free-ranging bottlenose dolphins living near Sarasota were analyzed for PIV antibodies. Because only 1 positive control animal had been identified, a conservative interpretation of ELISA results was adopted. Samples with an OD_405_ ratio >1.0 (seropositive) contained an antibody level at least as high as the positive control’s highest antibody level during the time of PIV infection. Samples with an OD_405_ ratio of 0.0 (seronegative) contained an anti-PIV antibody level that was less than or equal to the negative control. OD_405_ ratios >0 and <1 were categorized as inconclusive. Midrange values were used to compare mean OD_405_ ratios by population location, age, and sex. Midrange values were also used to assess changes within 1 animal over time (see Methods, PIV Seroconversion).

Descriptive statistics were used to determine the prevalence of PIV-seropositive, -seronegative, and -inconclusive animals among the 2 study populations. To assess the clinical relevance of PIV exposure among presumably healthy dolphins, mean values of hematologic and serum biochemical inflammatory indicators (leukocyte counts and ESR) were subsequently compared between PIV-seropositive and -seronegative animals in the MMP 2006 population by using analysis of covariance (ANCOVA) with a general linear model to control for varying numbers of samples, age, and sex of animals. Age and sex were controlled covariates because of the previously documented effects of age and sex on healthy, normal reference ranges in dolphins (*16*). A type I sum of squares p value was used to determine significance. Mean comparisons were reported by using least squares means when controlling for covariates.

PIV-antibody levels were compared between healthy MMP dolphins in San Diego and free-ranging dolphins living near Sarasota. Differences in age and sex between the 2 study populations were analyzed by using a general linear model (PROC GLM; CLASS population; MODEL age = population; MEANS population) and a Mantel-Haenszel χ2 test, respectively. Mean OD_405_ levels were compared by population, age, and sex (controlling for age due to identified differences in ages between the 2 populations) by using ANCOVA with a general linear model to control for varying numbers of samples by animal (PROC GLM Overview, SAS Online Doc, Version 8, SAS Institute, Inc.).

## Results

### Positive Control Case

**Figure 1:**
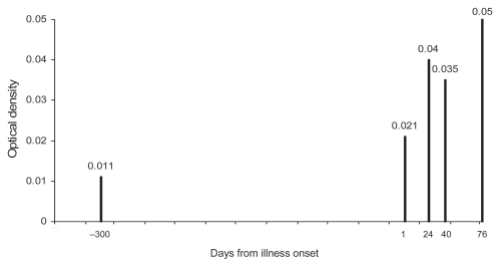
Sample series of parainfluenza virus (PIV) antibody optical density in the positive control bottlenose dolphin (*Tursiops truncatus*) during time of respiratory illness and multiple positive viral isolations from grossly affected lung**.**

PIV serum antibody OD_405_ levels were determined in a dolphin from which TtPIV-1 was successfully isolated from antemortem and postmortem lung samples. Low PIV antibody levels appeared to be present 300 days before illness, and rising antibody levels were detected during the course of TtPIV-1–associated respiratory illness (Figure 1).

### PIV Seroconversion

**Figure 2:**
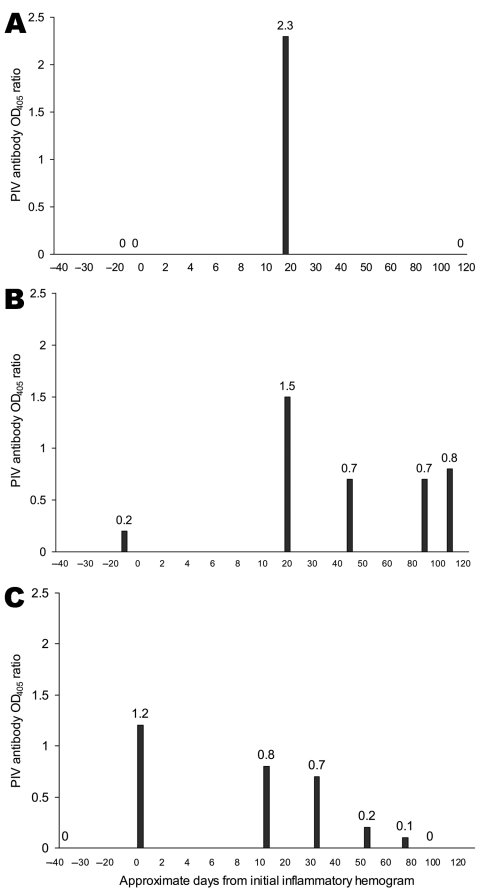
Evidence of active parainfluenza virus (PIV) infection in bottlenose dolphins (*Tursiops truncatus*) with inflammatory hemograms or clinical illness. A) PIV antibody optical density at 405 nm (OD_405_) ratios in a 3-year-old female bottlenose dolphin; B) PIV antibody OD_405_ ratios in a 26-year-old male bottlenose dolphin; C) PIV antibody OD_405_ ratios in a 22-year-old male bottlenose dolphin.

PIV antibody levels were determined in 588 serum samples collected during 1999–2006 from 58 selected MMP bottlenose dolphins before, during, and after an inflammatory hemogram similar to that of the positive control. Within this sample set, 22 dolphins were identified that seroconverted within a 3-month period (examples, Figure 2, **panels A–C**). Eleven (50%) of the dolphins that seroconverted were female; median age was 22.2 years (range 0.3–43 years).

As expected because of the selection criteria for suspected cases, dolphins with PIV seroconversion had a neutrophilic leukocytosis (18, 81.8%) or high ESR (16, 72.7%). Other clinicopathologic abnormalities that were present in at least half of the case dolphins included hyperglobulinemia, monocytosis, thrombocytosis, and high alanine aminotransferase (ALT) levels (Tables 1, 2).

**Table 1:** Frequencies of abnormal hematologic and inflammatory indicator values in bottlenose dolphins (*Tursiops truncatus*) within  60 d before and 30 d after PIV antibody seroconversion (n = 22)*

Blood variable	Adult dolphin reference range	Median value (range), cases	No. (%) cases below reference range	No. (%) cases above reference range
Leukocytes, cells/μL	4,275–10,089	12,950 (5,700–38,800)	2 (9.1)	18 (81.8)
HCT, %	38–46	39 (29–50)	11 (50.0)	0
Platelets, cells/μL	55,000–143,000	116,000 (25,000–333,000)	6 (27.3)	11 (50.0)
Neutrophils, cells/μL	2,737–7,570	9,750 (3,760–36,470)	2 (9.1)	18 (81.8)
Lymphocytes, cells/μL	270–1,500	1,425 (290–5,210)	3 (13.6)	7 (31.8)
Monocytes, cells/μL	0–576	300 (0–3, 080)	NA	11 (50.0)
Eosinophils, cells/μL	78–1,792	900 (0–3,000)	8 (36.4)	3 (13.6)
ESR, mL/60 min	0–18	22 (1–131)	NA	16 (72.7)
Iron, μg/dL	92–300	193 (22–1,036)	8 (36.4)	9 (40.9)

*PIV, parainfluenza virus; HCT, hematocrit; NA, not available; ESR, erythrocyte sedimentation rate.

**Table 2:** Frequencies of abnormal serum biochemical values in bottlenose dolphins (*Tursiops truncatus*) within 60 d before and 30 d after PIV antibody seroconversion (n = 22)*

Blood variable	Adult dolphin reference range	Median value (range), cases	No. cases below reference range (%)	No. cases above reference range (%)
Protein, g/dL	6.2–7.6	7.1 (5.7–9.6)	4 (18.2)	12 (54.5)
Albumin, g/dL	3.9–4.9	4.2 (3.4–5.1)	6 (27.3)	2 (9.1)
Globulin, g/dL	2.1–3.1	3.0 (1.7–5.0)	2 (9.1)	12 (54.5)
Glucose, mg/dL	85–144	110 (70–214)	7 (31.8)	9 (40.9)
Sodium, mEq/L	153–159	156 (127–169)	8 (36.4)	0
Chloride, mEq/L	115–125	118 (109–134)	12 (54.5)	0
Potassium, mEq/L	3.5–4.1	3.8 (3.0–7.5)	5 (22.7)	10 (45.5)
Calcium, mg/dL	8.3–9.7	9.1 (6.6–10.8)	3 (13.6)	9 (40.9)
BUN, mg/dL	36–59	48 (29–76)	8 (36.4)	6 (31.8)
Creatinine, mg/dL	1.2–2.0	1.3 (0.7–2.4)	7 (31.8)	4 (18.2)
Uric acid, mg/dL	0.0–0.7	0.2 (0.0–1.8)	NA	8 (36.4)
LDH, U/L	270–494	399 (247–1672)	0	10 (45.5)
AST, U/L	118–398	228 (111–987)	0	9 (40.9)
ALT, U/L	13–54	33 (15–541)	0	11 (50.0)
GGT, U/L	21–48	39 (18–876)	0	10 (45.5)
Carbon dioxide, mEq/L	17–28	24 (9–34)	3 (13.6)	4 (18.2)
Inorganic phosphate, mg/dL	3.9–5.9	5.1 (3.2–7.5)	6 (27.3)	9 (40.9)
Alkaline phosphatase, U/L	158–556	240 (36–1070)	8 (36.4)	3 (13.6)
Cholesterol, mg/dL	153–262	202 (115–382)	9 (40.9)	5 (22.7)
Triglyceride, mg/dL	11–175	95 (27–667)	0	8 (36.4)
CPK, U/L	51–183	122 (24–596)	5 (22.7)	9 (40.9)

*PIV, parainfluenza virus; BUN, blood urea nitrogen; NA, not available; LDH, lactate dehydrogenase; AST, aspartate aminotransferase; ALT, alanine aminotransferase; GGT, gamma-glutamyl transpeptidase; CPK, creatine phosphokinase.

Of 22 dolphins that seroconverted, 5 (22.7%) maintained normal behavior and appetite, and 17 (77.3%) had at least 1 clinical sign. The most commonly reported behavioral abnormalities were decreased appetite (12, 54.5%) and lethargy (10, 45.5%). Veterinary observations also included respiratory (7, 31.8%) and ocular (5, 22.7%) clinical signs (Table 3). Respiratory signs included ventral left lung consolidation, pleuritis, tachypnea, coughing, and abnormal blowhole fluid. Ocular clinical signs were reported as bilateral or unilateral blepharospasm, intermittent squinting, or corneal opacities. Other clinical signs were diffuse, miliary skin or mucosal lesions (2 dolphins) and orange-yellow liquid feces and excessive flatulence (2 dolphins).

**Table 3:** Clinical signs noted in selected bottlenose dolphins (*Tursiops truncatus*) within 60 d before and 30 d after PIV antibody seroconversion and an abnormal hemogram (n = 22)*†

Animal	Clinical signs	Decreased appetite	Lethargy	Respiratory signs	Ocular signs	Epidermal signs	GI signs
A							
B	X				X		X
C							
D	X		X				
E	X	X		X	X		
F	X	X					
G	X	X	X				
H	X	X	X		X	X	
I	X	X	X	X			
J	X	X	X	X			
K	X	X			X		X
L							
M							
N	X		X	X	X		
O							
P	X	X	X				
Q	X						
R	X		X				
S	X	X	X	X			
T	X	X		X			
U	X	X	X			X	
V	X	X		X			
Total	17 (77.3%)	12 (54.5%)	10 (45.5%)	7 (31.8%)	5 (22.7%)	2 (9.1%)	2 (9.1%)

*PIV, parainfluenza virus; GI, gastrointestinal. †No viruses were cultured from clinical samples collected from these animals, with the exception of the positive control dolphin, and 2 dolphins had culture-confirmed bacterial pneumonia concurrent with PIV seroconversion.

Of 13 dolphins that had clinical signs and recovered, the average duration of clinical illness was 9.8 days (range 1–40 days). Four dolphins had PIV antibody seroconversion within 30 days of death, but the presence of TtPIV-1 was not confirmed on virus culture from any animal tissues upon necropsy except from the positive control dolphin. Of these 4 dolphins, 2 died from bacterial pneumonia confirmed by laboratory culture and histologic examination, and 1 had mild to moderate growth of *Candida glabrata* from lung samples. All 4 animals had a mild to moderate tracheitis or laryngitis not explained by finding intralesional bacterial or fungal infection upon histopathologic examination.

### Population Seroprevalence

Comparisons of age and sex distribution among the 2 dolphin populations are provided in Table 4. The median age of dolphins was 15.5 years (range 0.2–49.2). Sex was determined for 110 dolphins; of these, 50% were female.

**Table 4:** Comparisons of age, sex, and PIV antibody levels among 2 healthy bottlenose dolphin (*Tursiops truncatus*) populations (n = 114)

Descriptor	Free-ranging dolphins, Sarasota, Florida (n = 56)	Managed dolphins, San Diego, California (n = 58)	p value
Mean age, y	11.5	20.7	<0.0001
Sex, %			
Female	45.6	54.7	0.3
Male	54.4	45.3	
Mean PIV antibody OD_405_ ratio*	0.42	0.36	0.6
PIV result, %			0.5
Negative	33.9	25.9	
Equivocal	58.9	58.6	
Positive	7.1	15.5	

*PIV, parainfluenza virus; OD_405_, optical density at 405 nm.

Of 114 clinically healthy dolphins tested for PIV antibodies, 13 (11.4%) were positive, 34 (29.8%) were negative, and 67 (58.8%) were inconclusive (0 <OD_405_ ratio <1.0). Mean and median PIV OD_405_ ratios were 0.4 (standard deviation = 0.4) and 0.2 (range 0.0–2.8), respectively. There were no significant differences in OD_405_ ratio by age (p = 0.2) or sex (females = 0.33, males = 0.43, p = 0.3). When Florida free-ranging healthy dolphins were compared with San Diego managed healthy dolphins, there were no significant differences in mean PIV OD_405_ ratios (p = 0.6) or percentages of animals categorized as negative, positive, or inconclusive (Table 4; p = 0.5). Among the 2006 MMP healthy dolphin serosurvey population, no significant differences were identified when hematologic indicators of inflammation among healthy PIV antibody–positive dolphins were compared with PIV antibody–negative dolphins (Table 5).

**Table 5:** Comparisons of mean inflammatory indicator values between bottlenose dolphins (*Tursiops truncatus*) seropositive or seronegative for parainfluenza virus antibodies, July–December 2006*

Blood variable, cells/μL	Least squares means, TtPIV1 seropositive (n = 9 animals)	Least squares means, TtPIV1 seronegative (n = 15 animals)	p value
Leukocytes	6,969	7,602	0.23
HCT	41.6	40.3	0.25
Lymphocytes	1,357	1,379	0.93
Monocytes	141	133	0.89
Neutrophils	4,389	5,189	0.05
60-min ESR	10.5	10.2	0.93

*TtPIV1, *T. truncatus* parainfluenza virus type 1; HCT, hematocrit; ESR, erythrocyte sedimentation rate.

## Discussion

Using an indirect dolphin-specific antibody ELISA, we demonstrated an increase in PIV serum antibodies during culture-confirmed TtPIV-1 respiratory illness in an adult bottlenose dolphin. Although ELISA has been recognized as the most sensitive indicator of PIV infections in human populations (*9*), virus isolation and genotyping are needed to confirm which type of PIV is associated with an infection (*10*). As such, antibody ELISA results in our study were interpreted as dolphin immune responses to PIV or a closely related virus (e.g., a mumps-like virus).

We report 21 additional dolphins in which PIV antibody seroconversion occurred within 3 months of an abnormal hemogram similar to that of the positive control animal during 1999–2006. Approximately 23% of these dolphins did not have overt clinical signs, indicating that PIV infections may affect hematologic values without affecting animal behavior. Further, no significant differences in inflammatory indicators were identified when PIV antibody–seropositive and –seronegative animals were compared in our cross-sectional serosurvey of healthy animals. Subclinical BPIV-3 infections are frequent in cattle populations (*17*). In a case-control study comparing acute- and convalescent-phase serum samples among calves with respiratory disease and calves that were clinically normal, the incidence of BPIV-3 seroconversion was actually higher in clinically normal calves (*18*).

In our survey involving dolphins that seroconverted within 3 months of an abnormal hemogram, clinical signs were most often nonspecific and limited to lethargy and decreased appetite lasting an average of 9–10 days. Of animals that seroconverted, 32% had respiratory clinical signs, and 3 of 4 animals that died within 30 days of seroconversion had intralesional bacterial or fungal pathogens in lung tissue. Further evidence of primary PIV infections in animals that died from bacterial or fungal pneumonia was inflamed laryngeal or tracheal tissue without intracellular bacterial or fungal pathogens. Despite confirmed bacterial or fungal pneumonia in these animals, pathologists’ interpreted the tracheitis and laryngitis to be of possible viral origin. In terrestrial mammals, PIV most commonly affects the upper and lower respiratory tract (*1*–*3*,*9*), and frequent conditions include tracheitis and laryngitis. Further, PIV infections are commonly associated with bacterial or fungal coinfections in terrestrial mammals (*19*–*21*).

In our study, nonrespiratory signs associated with PIV seroconversion involved the ocular, epidermal, and gastrointestinal systems. Additionally, 50% of dolphins with PIV seroconversion had high ALT levels not associated with medications, indicating potential hepatic involvement. Similarly, nonrespiratory clinical signs reported in a study involving 46 human patients with PIV virus infections included conjunctivitis, exanthema, oral mucosal lesions, diarrhea, and increased levels of transaminases (*22*).

Lacking in all active case dolphinss, except the positive control, was culture of PIV from clinical or postmortem samples. In humans, reported HPIV-3 viral culture success rates from clinical samples can range from 42% to 50% (*23**,**24*), and detecting PIV by looking for CPE is not considered reliable or useful (*9*). The diagnostic laboratory we routinely used to culture viruses from MMP samples relied primarily upon CPE in monkey kidney cell lines over multiple passages. To improve the likelihood of isolating PIV during active infections in bottlenose dolphins, diagnostic workups should include PCRs and indirect immunofluorescent-antibody assays (IFAs), as these tools have proven effective for rapidly identifying PIV cases in other species (*25*,*26*).

In our seroprevalence study involving 114 clinically healthy dolphins, we demonstrated that 11.4% had PIV antibodies at least as high as our positive control, and 70% had PIV antibodies higher than our negative control. No significant differences in PIV antibody levels were found when comparing dolphin location, age, or sex. Similar findings regarding equivalent PIV exposures by age, sex, and geographic location have been reported in humans (*9*) and cattle (*27*). High rates of PIV infection among these populations support incomplete immunization with infections and high rates of reinfection throughout life (*9*). Serologic studies have demonstrated common PIV exposure among wild hooved species in Alaska (67%) (*28*), the central Italian Alps (17%) (*29*), Argentina (43%) (*30*), Alberta (49%) (*31*), South Africa (25%) (*32*), and Quebec (82%–84%) (*33*).

The primary limitation of our study was interpretation of PIV antibody levels based upon 1 positive control. To compensate for the limitation of positive controls among marine mammal samples, we applied conservative definitions for positive and negative ELISA results and tested for significant changes in antibody levels in the same animal over time. Use of PCR and IFA as standard assays on prospective samples will help to increase the number of positive controls for future studies (*22*).

TtPIV-1 is a novel virus most closely related to bovine PIV3 (BPIV-3) (*4*), and attenuated BPIV-3 has been demonstrated as a safe and effective vaccine against human PIV3 (HPIV-3) in human populations (*34**,**35*). Given the genetic similarity of TtPIV-1 to BPIV-3 and HPIV-3, TtPIV-1 may provide therapeutic benefit to human populations.
